# Patterns of pulmonary dysfunction in asbestos workers: a cross-sectional study

**DOI:** 10.1186/1745-6673-5-12

**Published:** 2010-06-03

**Authors:** Belayneh A Abejie, Xiaorong Wang, Stefanos N Kales, David C Christiani

**Affiliations:** 1 University of California San Francisco School of Medicine, Fresno Medical Education Program, Fresno, CA, USA; 2 School of Public Health and Primary Care, The Chinese University of Hong Kong, SAR, China; 3 Harvard School of Public Health, Boston, MA, USA

## Abstract

**Background:**

Restrictive patterns of pulmonary function abnormalities associated with asbestos exposure are well described. Studies are less consistent, however, regarding the association of asbestos inhalation with airway dysfunction and obstructive impairment.

**Methods:**

We compared pulmonary function test results between 277 chrysotile exposed workers (22% non-smokers) and 177 unexposed controls (50.3% non-smokers). Information on exposure and smoking were collected using a standardized questionnaire. Standardized spirometric and DCLO Measurement methods were utilized. CXRs were read based on ILO pneumoconiosis guidelines.

**Results:**

Asbestos exposed subjects had significantly reduced FVC, FEV1, FEV1/FVC and DLCO. Restricting the analysis to non-smokers, asbestos workers still had about 3% lower FEV1/FVC ratio than controls, but this difference did not reach statistical significance. Among exposed workers, the presence of radiographic evidence of asbestosis further lowered FVC and DLCO but not FEV1/FVC compared to asbestos exposure without radiographic asbestosis. Additionally, smoking asbestos workers had significantly lower DLCO compared to non-smoking workers.

**Conclusion:**

Asbestos exposure, especially when radiographic evidence of interstitial fibrosis from asbestosis is present, leads to significant decreases in FVC, FEV1 and the DLCO. However, asbestos exposure alone is not significantly associated with a reduction of the FEV1/FVC. Smoking-asbestos workers had significantly lower DLCO than their non-smoking counterparts. Whether asbestos interacts with smoking additively or synergistically on DLCO needs further investigation. Similarly, further studies are needed to assess the progression and clinical significance of asbestos induced airway dysfunction.

## Introduction

The association of a restrictive pulmonary function with interstitial lung disease is well described [1-12]. However, the results of studies examining obstructive airway impairment in asbestos- exposure are not entirely consistent. Such investigations of airway function have been conducted in animal models, clinical series, and epidemiological surveys.

In 1982 Begin observed small and large airway disease in sheep with tracheal installation of high concentrations of chrysotile asbestos [13]. He further demonstrated that asbestos airway disease appears to be dose dependent [14]. In 1985, Filipenko et al found thickened membranous and respiratory bronchioles in Guinea Pigs [15]. Similarly, Bellis observed small air way lesions in lung autopsies [16]. Dumortier reported small airway pathologic changes in Guinea pigs after amosite exposure in 1990 [17]. However, whether asbestos can induce clinically significant obstruction in non-smoking human populations remains somewhat controversial. Additionally, because occupational exposure is often to mixed-mineral dust, rather than only to asbestos, the ability to extrapolate from animal studies to humans is limited.

Harless observed that chrysotile exposed workers developed abnormal FEF25-75 and nitrogen washout curves [18]. Consequently, Rodriguez-Roisin and his colleagues found flow volume curve abnormalities suggestive of small air way lesions [19]. Similarly, Begin found evidence of diminished flows at low lung volumes in non-smoking chrysotile workers [20], and Becklake observed an obstructive pattern of reduction in spirometry in groups with high dust exposure[21]. Later, Griffith et al demonstrated airway disease in a non-smoking cohort of asbestos workers [22]. Kilburn and Warshaw observed a reduction in FEV1, FEV1/FVC ratio, and an increase in RV/TLC, an obstructive pattern [23]. Wang et al showed significant decrease in FEF25-75% in older asbestos workers [12].

However; earlier studies [12,24-31] did not support the relationship of asbestos exposure to obstructive lung dysfunction. Similarly, in 1994, Miller et al did not observe strong evidence for obstructive impairment on 2611 participants (20% non-smokers) [32]. Still other studies have shown mixed PFT abnormalities [33-35]. Furthermore, in most of the studies, especially those conducted before the mid-nineties, either small sample size and/or the effect of smoking were limitations making interpretation difficult. Other areas of concern in most of the studies included the use of FEF, a less stable measure of obstruction[12,22,33,36], incomparable or absent control groups, [18,23,32], lack of DLCO measurement [32,33], single chest -x-ray reader [23], and use of unadjusted FEV1/FVC and RV/TLC ratios[23]. To provide additional information, we compared the pattern of pulmonary dysfunction in asbestos workers using spirometric and DLCO measurements in a relatively large groups of chrysotile exposed subjects and controls without asbestos exposure.

## Methods

### Study Population

As a part of a study on the respiratory health status of dust exposed workers, chrysotile factory workers were surveyed in 1989. The workers came from a factory in which asbestos textile products were manufactured in Southwest China. Their pulmonary examination included clinical evaluation, chest radiography, and spirometry and diffusion capacity (DLCO) measurements. Subject selection was restricted to male workers with direct asbestos exposure for at least 2 years, but no overt neuromuscular and clinical cardiopulmonary disorders other than pneumoconiosis at the time of survey. Invitations for participation covered all current workers, and retired workers who were living close to the asbestos factory. Retired workers living far from the factories were not included for logistic reasons. Women were not included because they comprised a very small number. Study subjects were not exposed to other fibers or dust except asbestos.

Control groups were drawn from employees of the electronic industry located in the same geographic area as the asbestos factory. Selection was restricted to male workers with at least 2 years work history, no history of asbestos or any other dust exposure, and no overt cardiopulmonary and neuromuscular problems. The study was approved by the Human Subjects Committee of the West China University Medical School.

### Exposure Assessment

The factory was established in 1950. Since the 1970s some engineering control measures were in place, but in most cases the area sample concentration range still exceeded 2 mg/m^3^, the Chinese maximum allowable concentration at that time. Workers did not use personal protective equipment. During their stay in the plant, employees changed job types frequently and did not hold the same job title for long period of time. Therefore, the individual cumulative duration of work in exposed areas was used as surrogate measure of total asbestos exposure.

### Clinical Evaluation

Using a Chinese standardized respiratory questionnaire, which was based on Medical Research Council Questionnaire [37], face- to- face interviews of both exposed and control groups was conducted by two physicians. Information was gathered on demographic data, occupational history, smoking habits and respiratory symptoms. Special attention was given to job title and beginning and end dates at each job in occupational history. Smoking was quantified in pack years and also categorized in to 3 qualitative groups defined as follows. Current smokers were those who were currently smoking or had quit smoking less than 3 months before the time of interview; ex-smokers as those who quit smoking at least 3 months prior to the interview and non-smokers as those who had never smoked more than 20 packs of cigarettes in their life time or no more than 1 cigarette per day for one or more years. Pack years were defined as the number of packs (one pack = 20 cigarettes) multiplied by the number of years smoked.

### Radiographic Evaluation

Posterior-Anterior (PA) chest-x-rays (CXR) on full inspiration and standing position were done at least once for each asbestos worker and were read by panels for pneumoconiosis and emphysema. Panel members include pulmonologists, radiologists and occupational health experts. Readers were blinded to PFT values and the CXR findings were based on the consensus of at least two experts. The 1986 Chinese Roentgeno-Diagnositc criteria of pneumoconiosis, established based on the 1980 international labor organization (ILO) classification of pneumoconiosis, were used to grade the severity of asbestosis. Stage 0, I, and II correspond to ILO stages (0/-to 1/0), (1/1 to 2/3) and (3/2 to 3/+) respectively. Stage III represents ILO large opacities with categories A, B, and C. Radiographic asbestosis was defined as perfusion densities stage I (1/1) or greater in persons with a history of asbestos exposure. There is a good agreement between the Chinese Roentgeno-Diagnosis criteria of pneumoconiosis and ILO CXR system [38]. Emphysema was diagnosed and graded radiographically as none, mild, moderate or severe by panel member consensus.

### Spirometric and DCLO Measurements

A 9-L water- sealed spirometer (Godart Pulontest, NV, The Netherlands) was used to measure FVC and FEV1 following ATS guidelines [39]. Participants did not smoke for at least one hour before the test. At least three acceptable efforts were obtained in each participant while wearing nose clips in standing position. Care was taken to maintain expiration for at least 6-seconds or until flow plateau was observed. The largest values of FEV1 and FVC were chosen for analysis. Single breath diffusion capacity for carbon monoxide (DLCO) test was performed based on Epidemiology Standardization Project protocol [40]. Subjects were in sitting position during the test and the breath hold time was 10-seconds. For subjects with FVC of 2 or more liters (L), the washout volume was 1 L and for those with FVC of less than 2 L, the washout volume was 0.5 L. A pulmonary gas analyzer (GC-1, Shanghai, China) was used for gas analysis. DLCO was calculated using inspired volume, breath hold time, and CO and helium concentrations. Measured values (except FEV1/FVC ratios) corrected for body temperature, ambient pressure and saturated water vapor were expressed as the percentage of predicted values calculated with equations that considered age, height and gender derived from the Chinese general population. The same team of technicians conducted the tests in both the exposed and control groups using same equipment and procedures. Although PFT technicians were not blinded to exposure status (because testing was conducted on worksite), they were not aware of the clinical and radiographic characteristics of each participant. Similar to Ohar et al [41], mutually exclusive predictive value percentages were used to define PFT patterns as follows. Normal: FVC ≥80%, and FEV1/FVC ≥70%; Restrictive: FVC < 80% and FEV1/FVC ≥70%; Obstructive: FVC ≥80% and FEV1/FVC < 70%; and Mixed: FVC <80% and FEV1/FVC <70%.

### Statistical Methods

The mean values of baseline characteristics were obtained from the SAS proc means procedure (SAS--9 version). Multiple regression techniques were utilized to analyze the relationships of exposure and other independent variables with pulmonary function test values. With regard to smoking, pack-years rather than yes/no was included in the regression models. In all analyses, a p value less than 0.05 (two sided) was considered significant. SAS software (Version 9.1, Cary, NC) was used for all statistical data analyses.

## Results

Two hundred seventy seven asbestos workers and 177 control subjects were included in the study (Table 1). The participation rates for exposed and control subjects were not different: 77% and 80% respectively. The asbestos workers were significantly older than the controls. Smoking was more frequent among asbestos workers, and they also had smoked a greater number of pack years. Among the asbestos workers, 36% had radiographic changes consistent with emphysema, 31% with asbestosis, and 15% had CXR findings consistent with both asbestosis and emphysema.

**Table 1:** Basic Characteristics

Variable	Exposed (N = 277)	Controls (N = 177)	P-value
Age, mean(SD)	55.1 (12.0)	37.2 (10.1)	.0001
Height, mean(SD)	162.5 (5.9)	167.0 (5.8)	.0001
Exposure year, mean(SD)	16.7 (9.3)		
Pack year, mean(SD)	27.9 (18.3)	10.8 (8.3)	.0001
FEV1, mean(SD)	96.6 (22.0)	103.9 (13.9)	.0001
FVC, mean(SD)	96.3 (17.0)	102.6 (12.4)	.0001
FEV1/FVC, mean(SD)	71.3 (12.1)	78.3 (8.0)	.0001
DLCO, mean(SD)	97.1 (21.6)	124.6 (18.8)	.0001
Ever smokers (%)	78.0	49.7	
Asbestosis (%)	30.7		
Emphysema (%)	35.7		
Asbestosis emphysema (%)	15.2		

**Figure 1:**
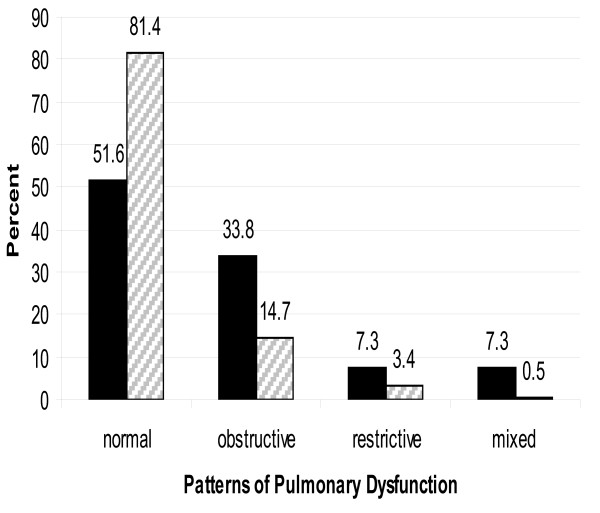
**Patterns of Pulmonary Dysfunction in Exposed and Control Groups**. Exposed (N = 277). □ Controls (N = 177).

As shown in figure 1, more than 80% of the controls had normal pulmonary function compared to only half of the asbestos workers had normal pulmonary function. Consequently, the proportions of subjects with obstructive, restrictive and mixed patterns of pulmonary dysfunction in the exposed group were all higher than the corresponding proportions in the control group.

Although the PFT values except the FEV1/FVC ratio were adjusted for age and height, we also included age in the regression analysis as there was significant age difference between the exposed and control groups (Table 2). Because we were interested in examining the effect of mere asbestos exposure (without CXR evidence of asbestosis) on patterns of pulmonary, we excluded patients with radiographic asbestosis from the model. After accounting for age and smoking, asbestos exposure was significantly associated with restrictive pattern of pulmonary dysfunction and decreased DLCO. However, asbestos exposure was not significantly associated with FEV1/FVC ratio. Similar results were found when FEV1, FVC, and DLCO were regressed on exposure status and pack year without including age in the model (not shown).

**Table 2:** Regression Analysis: Control and Exposed Groups without Asbestosis (N = 369)

	FEV1	FVC	FEV1/FVCa	DLCO
Age	0.09 (0.08)†	0.11 (0.07)	-0.40 (0.05)*	-0.20 (0.10)*
Exposure	-6.69 (2.62)*	-5.72 (1.81)*	0.25 (1.26)	-19.30(2.71)*
Pack Year	-0.14 (0.07)*	-0.06 (0.05)	-0.07 (0.04)*	-0.173(0.07)

† = coefficient (standard error) a = adjusted for height * = significant (p < .05)

As shown in Table 3, when PFT values were regressed on exposure status in non-smokers only, asbestos exposure was significantly associated with low FEV1, FVC and DLCO percent predicted values after accounting for age. In addition, the results indicate that non-smoking asbestos workers had close to 3% less FEV1/FVC ratios compared to non -smoker control workers of similar age and height. This last relationship, however, was not statistically significant. For FEV1, FVC and DLCO, the results were similar when age was excluded from the regression model as the PFT values were adjusted for age (analysis not shown).

**Table 3:** Regression Analysis: Non-Smokers (N = 130)

	FEV1	FVC	FEV1/FVC a	DLCO
Age	0.22 (0.12)†	0.18 (0.10)	-0.38 (0.07) *	0.12 (0.17)
Exposure	-11.67(3.69)*	-6.58 (3.14)*	-2.79(2.07)	-31.87(5.11)*

† = coefficient (standard error) a = adjusted for height * significant(p < .05)

To compare the effect of radiographic asbestosis on PFT values as opposed to asbestos exposure (without asbestosis), we performed regression analysis of PFT values on asbestos exposed subjects only (Table 4). We removed patients with radiographic emphysema in this analysis to avoid the possible confounding effect of emphysema. As expected, individuals with radiographic asbestosis had significantly lower FVC and DLCO values than those asbestos exposed individuals without asbestosis. However, there was no significant difference in the FEV1/FVC ratio between these two groups.

**Table 4:** Regression Analysis: Exposed Subjects without Emphysema (N = 175)

	FEV1	FVC	FEV1/FVCb	DLCO
Asbestosis	-1.85 (3.46)†	-5.66(2.91)*	0.75 (1.6)	-9.76 (3.93)*
Pack Year	-0.14 (0.08)	-0.03 (0.06)	-0.10 (0.04)*	-0.21 (0.09)*

† = coefficient (standard error) b = adjusted for age & height * = significant(p < .05)

Finally, to examine the effect of smoking per see on DLCO and FVC values, we performed regression analysis on exposed subjects who do not have radiographic asbestosis or emphysema (Table 5). As expected, pack-years of smoking was significantly associated with FEV1 and FEV1/FVC ratio. Similarly, the pack-years variable was significantly associated with DLCO. Furthermore, pack-years was negatively related to FVC, although not statistically significant.

**Table 5:** Regression Analysis: Exposed groups without asbestosis and emphysema (N = 132)

	FEV1	FVC	FEV1/FVCa	DLCO
Pack Year	-0.21 (0.08)†*	-0.12(0.08)	-0.09 (0.05)*	-0.25(0.07)*

† = coefficient (standard error) a = adjusted for age & height * = significant(p < .05)

## Discussion

Our study supports, that asbestos exposure, with or without radiographic asbestosis, contributes to obstructive airway impairment. The proportion of asbestos exposed subjects with obstructive pulmonary impairment was about 2.5 times higher than that of the controls. However, caution should be exercised in the interpretation of our results, because different smoking habits may explain some of the difference as more than 80% of the participants in exposed group were smokers compared to 50% in controls. In a separate regression analysis, we found no significant difference in FEV1/FVC ratios between non-smoking asbestos workers and non-smoking controls, but asbestos workers still had almost 3% lower FEV1/FVC ratios compared to their corresponding controls (Table 3). We believe this difference did not reach statistical significance due to limits of our available sample size. Moreover, the FEV1/FVC ratio generally reflects large airways function, and the earliest asbestos lung lesions are peribronchiolar, and abnormalities in this anatomic region of the lung are not well-captured on standard pulmonary function testing [39,42]. In addition to smoking, another potential confounder was age. The asbestos workers were significantly older than the controls. However, we believe that we minimized any confounding by age. Age was adjusted for twice (first using PFT prediction equation, and again in multivariate regression analysis).

Our findings are in general agreement with past studies, finding excess obstruction among asbestos exposed workers, but remaining inconclusive as to how much of the effect is independent from smoking. Kilburn reported significant differences in FEV1/FVC and RV/TLC between non-smoking asbestos exposed subjects and controls in 1994[23]. However, his study was criticized for using unadjusted FEV1/FVC and RV/TLC ratios [43]. Several other studies showed similar results. For instance, Harless [18] demonstrated airflow obstruction in 23 heavily exposed male asbestos workers. Garcia-Closas, M and Christiani, DC reported mixed (restrictive-obstructive) patterns in a study of carpenters with pleural plaques [34]. Similarly, airway dysfunction has been reported in several other studies [18-20,22].

However, Miller did not observe significant differences in FEV1/FVC and FEF25-75% among non-smoking asbestos exposed subjects compared for duration of exposure in 1994[32]. Similarly, Sue et al reported that cigarette smoking, not asbestos, was the major contributing factor for the decline in FEV1/FVC ratio in asbestos-exposed workers in 1985[28]. Earlier studies in the 1970's did not support the claim that asbestos exposure was associated with airway dysfunction [24,26,27,29]. However, most of these studies had serious limitations such as the lack of unexposed controls and failure to control the effect of smoking. The strengths of our study included the use of unexposed controls from the same area and socioeconomic stratum, a detailed smoking history, and the analysis of airway dysfunction, and the use of standardized (ATS) pulmonary function testing and interpretation criteria.

The proportion of subjects with restrictive impairment in the exposed group was 2.2 times more than the corresponding proportion of subjects in the control group. The historical area sample concentrations, lack of exposure control measures in the company and the average duration of exposure (16.7 years) supports that the magnitude of asbestos exposure was high. Our study shows that asbestos exposure (without radiographic asbestosis) is significantly associated with decreased FVC, FEV1 and DLCO, consistent with previous studies [1-12]. The reduced FVC does not necessarily indicate volume loss as it could result from air trapping. In addition, the marked DLCO reduction in exposed subjects favors interstitial lung disease with alveolar involvement, since asbestos does not cause emphysema. Similarly, one may argue that pleural diseases might have contributed to the reduced FVC and FEV1. However, as Miller, and Garcia-Closas and Christiani pointed out, the association between discrete pleural diseases (plaques), and restrictive impairment is weak [32,34,35]. Given that pleural plaques are rare with in less than 20 year of exposure [ATS2004], and the average exposure of our study group was less than 11 years, pleural thickening is unlikely to explain our findings. Furthermore, the marked difference in DLCO again supports substantial early interstitial abnormalities that are not detected by plain radiographs. The proportion of subjects with a mixed pattern of pulmonary impairment in exposed subjects was more than 14 times greater than among the controls, which is also consistent with other previous findings[34].

As expected, workers with radiographic asbestosis had significantly lower FVC and DLCO values compared to other exposed workers. However, the two groups were similar in terms of FEV1/FVC ratio. This finding is consistent with those of Kilburn and Warshaw findings [23]. The reason for lack of significant difference in FEV1/FVC ratio between those asbestos exposed groups with and without radiographic asbestosis is not clearly understood. Some say airway dysfunction is not related to asbestos fiber burden[22]. However, others observed small airway dysfunction only in long-term exposure, [2,11,19] and still others claim that low cumulative exposures are less likely to produce airway abnormalities [5,27,44]. Other explanations include enhanced elastic recoil in asbestosis [ATS 2004] and increase lung radial traction by fibrosis[36].

Asbestos workers (without radiographic asbestosis or emphysema) who smoked had significantly lower DLCO, and not surprisingly lower FEV1 and FEV1/FVC ratio compared to asbestos workers who did not smoke. Among 21 studies, reviewed by Weiss, 11 showed an additive positive interaction between smoking and asbestos[45]. Similarly, Kilburn and Wright reported a synergistic effect of smoking with asbestos in insulators and Guinea pigs respectively [46,47]. However, Alfonso et al reported no significant interaction between asbestos and smoking[1].

Although the mechanism for asbestos related interstitial pulmonary diseases is well described, the pathogenesis of asbestos-related disease obstructive airway diseases is still unsolved. Begin et al reported peribrochial alveolitis, in high dose crysotile asbestos exposed sheep and fibrosis with obliteration and narrowing of the small airways in lung biopsy of three asbestos workers in 1982 and 1983 respectively [1,13,48]. Wright and Churg demonstrated sever diffuse airway pathology after studying necropsy of 36 asbestos miners and their matched controls in 1985[49]. Similarly, Filipenko et al demonstrated significantly thickened non-cartilaginous airways in amosite exposed guinea pigs in 1985[15]. On the other hand Griffin et al claimed that mineral-dust airway disease is irritant phenomenon based on individual susceptibility irrespective of dust burden[22].

Our study had several limitations: First, the asbestos workers were significantly older than the controls. However, age was adjusted for in both the predictive equations and our regression model. Second, unlike the radiographic panel experts, the PFT technicians were not blinded to the status of asbestos exposure. Other weaknesses include the lack of chest films in controls, the absence of pleural radiographic information in asbestos workers, and the lack of area or personal asbestos measurements for exposure assessment. Nonetheless, these limitations do not negate our findings of the lower pulmonary function among the asbestos exposed workers.

In conclusion our study showed that asbestos exposure with or without radiographic asbestosis is significantly associated with reduced DLCO and restrictive lung impairment. However, asbestos exposure was not significantly associated with reducedFEV1/FVC. Among the exposed workers, radiographic asbestosis was associated with lower FEV1, FVC and DLCO values, but was not associated with any further reduction in the FEV1/FVC ratio. Finally smoking-asbestos exposed subjects had significantly reduced DLCO compared to their non-smoking counterparts. Further investigation is needed to determine whether combined exposure to asbestos and smoking act in an additive or synergistic fashion in reducing lung function, and to assess the progression and clinical significance of asbestos-induced airway impairment.

## Competing interests

The authors declare that they have no competing interests.

## Authors' contributions

BA conceived the study hypothesis; conducted the data analysis; participated in the interpretation of results and drafted the paper.

XW developed the study design; managed the data collection; and participated in the analysis and interpretation of the results.

SK participated in the interpretation of results, paper writing and editing.

DC supervised the analysis, interpretation of results and paper editing; raised funding.

All the authors read and approved the final manuscript.
